# Fluorescent–Electrochemical–Colorimetric Triple-Model Immunoassays with Multifunctional Metal–Organic Frameworks for Signal Amplification

**DOI:** 10.3390/bios15060376

**Published:** 2025-06-11

**Authors:** Ning Xia, Chuye Zheng, Gang Liu

**Affiliations:** Henan Province Key Laboratory of New Opto-Electronic Functional Materials, College of Chemistry and Chemical Engineering, Anyang Normal University, Anyang 455000, China; 13850182553@163.com (C.Z.); liugang08215@163.com (G.L.)

**Keywords:** triple-model immunoassays, metal–organic frameworks, fluorescence immunoassay, electrochemical immunoassay, colorimetric immunoassay

## Abstract

Multimode immunoassays based on multiple response mechanisms have received great attention due to their capacity to effectively improve the accuracy and reliability of biosensing platforms. However, few strategies have been reported for triple-mode immunoassays due to the shortage of multifunctional sensing materials and the incompatibility of signal transduction methods in different detection modes. In this work, a fluorescent–electrochemical–colorimetric triple-mode immunoassay platform was proposed with Cu-based metal–organic frameworks (MOFs) as the signal labels. The captured Cu-MOFs were successfully decomposed under an acidic condition, leading to the release of numerous Cu^2+^ ions and 2-aminobenzene-1,4-dicarboxylic acid (NH_2_-BDC) ligands. The released NH_2_-BDC were determined by fluorescence titration. Meanwhile, the released Cu^2+^ were readily quantified by differential pulse voltammetry (DPV) and simply detected through the catalytic oxidation of chromogenic substrate 3,3′,5,5′-tetramethylbenzidine (TMB). Taking alpha-fetoprotein (AFP) as a model analyte, the designed triple-mode immunoassays showed good performances with the linear range of 10–200 pg/mL, 10–200 pg/mL, and 1–100 pg/mL for the fluorescent, electrochemical, and colorimetric modes, respectively. The proposed triple-mode biosensing platforms show great potential for the applications in disease diagnosis, since they can be easily extended to other bioassays by changing the targets and recognition elements.

## 1. Introduction

Biomarkers are a type of markers associated with cell growth and proliferation. In recent years, with the development of immunology and molecular biology technologies, more and more biomarkers have been discovered. They mainly exist in blood, urine, feces, tissues, cells, and hair [1,2]. The accurate determination of biomarkers can enable an exploration of pathogenesis at the molecular level, providing early warnings and an auxiliary basis for clinical diagnosis and helping to evaluate the damage in the early low-level stages of diseases. Developing advanced technologies for the accurate and sensitive detection of biomarkers is of great significance for disease identification, early diagnosis and prevention, and treatment evaluation. The current methods for monitoring the levels of biomarkers mainly include chemiluminescence, mass spectrometry, electrophoresis, and immunoassays [3,4]. Among them, immunoassays based on specific antigen–antibody interactions have been widely developed and applied for the analysis and detection of biomarkers in various biological samples due to their high sensitivity and specificity [5]. However, these techniques typically refer to a single-mode detection format, which is susceptible to various factors such as unstable experimental environments, non-standard experimental procedures, and unskilled operators, resulting in abnormal fluctuations in signal strength [6]. From this viewpoint, there is still a need to improve the accuracy and reliability of immunoassays.

Dual-mode adaptive immunoassays, such as colorimetric/fluorescent, colorimetric/electrochemical, and photothermal/colorimetric methods, have developed into attractive and promising technologies that can ensure reliable detection by combining different sensing mechanisms and cross-validation capabilities [6,7,8,9]. The dual-mode immunoassay is a type of analytical technique that can produce two detectable signals when an antibody binds to a target in a single reaction system. The results of dual-mode immunoassays allow for signal readout using two unique biosensing technologies, given their intelligent design. Although dual-mode immunoassays require more experimental steps compared to those with a single-mode detection format, their advantages are very attractive, such as an expanded detection range, improved detection diversity, and good cross validation by combining the advantages of various signal output modes. Generally speaking, the basic design of dual-mode immunoassays involves dual-functional nanomaterials, two different signal probes, and sample concentration methods such as magnetic separation or centrifugation [7]. This allows for the flexible use of two object-detection options in various analytical applications.

In order to further improve the accuracy and reliability and expand the linear range of sensing platforms, triple-mode biosensors have received great attention. For example, Zheng et al. prepared a multifunctional hybrid material named ethyl violet (EV)@NH_2_-MIL-88B(Fe) for the photoelectrochemical–fluorescent–colorimetric triple-mode detection of human papillomavirus 16 DNA with the assistance of CRISPR-Cas12a [10]. Gao et al. proposed a colorimetric/surface-enhanced Raman scattering/fluorescence triple-mode method for the rapid and selective detection of viral RNA by using AuNPs to adsorb DNA [11]. Meng et al. reported a photoelectrochemical–colorimetric–photothermal triple-mode immunoassay platform for the detection of prostate-specific antigen (PSA) by using multifunctional TiO_2_/ZIF-8/Cu(II) hybrids and alkaline phosphatase-labeled magnetic probes [12]. Very recently, Guo et al. developed a colorimetric–colorimetric–fluorescent triple-readout immunoassay platform for the detection of ochratoxin A with alkaline phosphatase as the signal label and Cu^2+^ as the trigger [13]. These triple-mode biosensing platforms can improve analytical accuracy and reliability due to the mutual validation in multiple detection modes, thus showing great potential in bioanalysis for the early diagnosis of diseases. Nevertheless, the reported triple-mode biosensors are far less than the proposed dual-mode platforms due to the lack of multifunctional sensing materials. Especially, few methods were proposed for triple-mode immunoassays.

Metal–organic frameworks (MOFs) are a class of porous crystalline materials which are formed by the assembly of metal ions/clusters and organic ligands. They have been widely employed in the field of adsorption and separation, heterogeneous catalysis, drug delivery, light harvesting, and chemical/biological sensing [14,15,16,17]. Both pristine MOFs and MOF-based composites have been successfully used as the signal labels of various immunoassays [18,19,20]. However, few works have addressed MOF-based triple-mode biosensing platforms. An elaborate selection of metal ions and organic ligands can endow MOFs with unique functionalities such as optical/electrochemical properties and catalytic activities. Redox-active metal ions or organic ligands serving as the precursors of MOFs can be directly determined by optical or electrochemical techniques. For example, taking advantage of the well-defined voltametric signal and catalytic activity of Cu^2+^, Cu-based MOFs have been used as redox probes and nanozymes for the development of sensing platforms [21,22]. In addition, based on the optical properties of organic ligands, MOFs can serve as the signal labels of colorimetric and fluorescence immunoassays. For instance, MOFs with 2-aminobenzene-1,4-dicarboxylic acid (NH_2_-BDC) as the fluorescent organic ligand have been applied for the design of colorimetric–fluorescence dual-mode immunoassays in combination with other nanomaterials for signal readout and amplification [23]. MOFs or metal nanoparticles modified on MOFs showing peroxidase-like activity can catalyze chromogenic reactions to produce colorimetric signals. Metal ions and small organic molecules serving as signaling reporters exhibit intrinsic advantages such as simple preparation, high scalability, good stability, and signal multiplicity. The structures of MOFs can be dismantled under an acidic or alkaline condition, leading to the release of fluorescence ligands and metal ions [23,24,25,26]. After the immunoreactions, a large number of metal ions or signaling molecules can be released and determined by different techniques, allowing for multiple-mode and signal-amplified detection. Therefore, the methods based on the assembly of metal ions and signaling molecules will be very sensitive and feasible for multiple-mode immunoassays. To the best of our knowledge, there are few reports on the design of triple-mode immunoassays with MOFs as signal labels.

Copper, a fundamental biological metal element and essential cofactor of many metalloenzymes, shows well-defined redox waves and high catalytic activities. Cu^2+^ and its complexes can be electrochemically reduced and can promote the oxidation of some reducing reagents or the reduction in H_2_O_2_ and O_2_ via redox cycling or catalytic reactions. The past few decades have witnessed a large number of reports on Cu^2+^ detection and copper-based biosensors with improved simplicity and sensitivity [21,27]. In this work, we proposed a triple-mode immunoassay platform by using Cu-BDC MOFs as the signal labels. The dissolution of Cu-BDC MOFs under an acidic condition resulted in the release of numerous NH_2_-BDC molecules and Cu^2+^ ions. The released NH_2_-BDC molecules emitted a strong fluorescence signal. Meanwhile, the released Cu^2+^ could be readily quantified by differential pulse voltammetry (DPV) and simply determined based on the catalytic oxidation of chromogenic substrate 3,3′,5,5′-tetramethylbenzidine (TMB) by H_2_O_2_. Therefore, a fluorescent–electrochemical–colorimetric triple-mode immunoassay platform was developed by determining the amounts of NH_2_-BDC and Cu^2+^ released from the multifunctional signal labels.

## 2. Materials and Methods

### 2.1. Chemical Reagents

Alpha-fetoprotein (AFP), anti-AFP, and biotinylated detection antibody (bio-Ab) were obtained from Linc-Bio Science Co., Ltd. (Shanghai, China). Prostate-specific antigen (PSA) and carcinoembryonic antigen (CEA) were ordered from Sangon Biotech Co., Ltd. (Shanghai, China). Recombinant streptavidin (rSA) was obtained from Prospec-Tany Technogene Ltd. (Ness-Ziona, Israel). Bovine serum albumin (BSA), thrombin, and NH_2_-BDC were provided by Sigma-Aldrich Company (Shanghai, China). Gibco fetal bovine serum was ordered from Thermo Scientific, Inc. (Shanghai, China). Carboxylated magnetic bead (MB) was ordered from Nanjing XFNANO Materials Tech Co., Ltd. (Nanjing, China). Polyvinyl pyrrolidone (PVP), N,N-dimethylformamide (DMF), Cu(NO_3_)_2_⋅3H_2_O, N-(3-(dimethylamino)propyl)-N′-ethylcarbodiimide (EDC), N-hydroxysulfosuccinimide (sulfo-NHS), and other reagents were ordered from Aladdin Reagent Company (Shanghai, China). To decompose Cu-BDC MOFs, 10 mM hydrochloric acid solution containing 10% DMF was used. All chemical reagents were of analytical grade and used without further purification. Ultrapure water (18.2 MΩ cm) was prepared using a purification system for the preparation of aqueous solutions.

### 2.2. Apparatus

Scanning electron microscopy (SEM) (Hitachi, Tokyo, Japan) equipped with an energy dispersive X-ray spectroscopy (EDS) was used to characterize the morphology and elemental composition of Cu-BDC MOFs. The crystal structure of MOFs was characterized by an Ultima III X-ray powder diffractometer (XRD) (Rigaku, Osaka, Japan). The Fourier transform infrared (FT-IR) spectra were collected on a NEXUS-470 spectrometer (Thermo Scientific, Inc., Waltham, MA, USA). Dynamic light scattering (DLS) and Zeta potential were measured on a Malvern Zetasizer Nano ZS model ZEN 3600 (Worcestershire, UK). Differential pulse voltammetry (DPV) measurement was conducted on a CHI660E electrochemical workstation (CH Instruments Inc., Shanghai, China) with a three-electrode system. A glass carbon electrode, a platinum wire, and a Ag/AgCl electrode were used as the working, auxiliary, and reference electrode, respectively. UV–vis absorption spectra were collected on a Cary 60 spectrophotometer (Santa Clara, CA, USA). Fluorescence data were collected on a Hitachi F-4600 fluorescence spectrometer (Hitachi High-Tech, Tokyo, Japan) with 5 nm excitation and emission slit width.

### 2.3. Preparation and Modification of Cu-BDC MOFs

For the preparation of Cu-BDC MOFs, NH_2_-BDC and Cu(NO_3_)_2_⋅3H_2_O were used as the organic ligand and metal material, respectively, and PVP was used as the stabilizer. First, 0.2 g of PVP was dissolved in 8 mL of DMF/ethanol mixed solvent (V/V = 1:1). This was followed by the addition of Cu(NO_3_)_2_⋅3H_2_O (24.2 mg) and NH_2_-BDC (5.4 mg) dissolved in 4 mL DMF. The mixture was sonicated for 30 min and then transferred to a Teflon-lined stainless-steel autoclave and reacted at 100 °C for 8 h. After cooling to room temperature, the Cu-BDC MOFs were centrifuged several times with DMF and ethanol to eliminate the residual surfactant or excess reagent. The obtained solid was finally dried under vacuum at 60 °C overnight.

The avidin–biotin system has been commonly used in immunoassays for the immobilization of biotinylated antibody. In this work, His_6_-tagged rSA was attached onto the surface of Cu-BDC MOFs via the metal coordination interactions, which can allow for the immobilization of biotinylated antibody with high avidity, specificity, and stability [28]. In brief, 0.5 mg Cu-BDC MOFs were dispersed in 1 mL of 20 mM phosphate-buffer solution (pH 7.4), followed by the addition of 50 μL of 1 μg/mL rSA. After being shaken at room temperature for 60 min, the rSA-modified Cu-BDC MOFs (Cu-BDC@rSA) were collected by centrifugation and washing and then dispersed in 1 mL of phosphate buffer. Then, 100 μL of bio-Ab (0.1 mg/mL) was added to the Cu-BDC@rSA suspension. After being shaken for 30 min, the detection antibodies were attached on the MOF surface through the avidin–biotin interactions. The resulting Cu-BDC@rSA-Ab conjugates were centrifuged, thoroughly washed with water, and then re-dispersed in 5 mL of phosphate buffer for use.

### 2.4. Modification of MB with Anti-AFP

The surface of carboxylated MB has abundant carboxyl active sites. Anti-AFP was attached onto the MB surface via the standard amino coupling reaction. In brief, 200 μL of carboxylated MB was diluted to 800 μL with 50 mM phosphate buffer (pH 6), followed by the addition of 0.4 M EDC and 0.1 M sulfo-NHS dissolved in 500 μL of phosphate buffer. After reacting for 15 min, the activated MB was separated by magnet and then incubated with 0.5 mL of 1 mg/mL anti-AFP for 2 h, followed by the addition of 100 μL of 10 μM BSA to seal the unreacted activated carboxyl groups. After incubation for 1 h, the resulting anti-AFP modified MB (denoted as AbMB) was washed three times and then re-suspended in 1 mL of phosphate buffer (20 mM, pH 7.4) for use.

### 2.5. Procedure for Triple-Mode Immunoassays

AFP was determined with the following procedures:

(i) The capture of AFP: A total of 20 μL of the prepared AbMB was mixed with a given concentration of AFP in 500 μL phosphate buffer (50 mM, pH 7.4) for 30 min.
(ii) The capture of Cu-BDC@rSA-Ab: After magnetic separation and washing three times with water, the AFP@AbMB was supplemented with 50 μL Cu-BDC@rSA-Ab conjugates to incubate for 30 min.
(iii) The dissolution of Cu-MOFs: The resulting Cu-BDC@rSA-Ab@AFP@AbMB conjugates were separated by magnet and washed three times with water. This was followed by the addition of 10 mM hydrochloric acid solution containing 10% DMF (25 μL) to release NH_2_-BDC and Cu^2+^ from the conjugates.
(iv) Fluorescence and electrochemical measurement: A total of 100 μL of 50 mM acetic acid buffer solution containing 0.1 NaCl was added to dilute the supernatant for the fluorescence assay and DPV measurement. The fluorescence spectra were collected with an excitation wavelength of 340 nm. The DPV responses were recorded from 0.25 to –0.05 V.
(v) Colorimetric assay: A total of 10 μL of 2.5 mM TMB and 10 μL of 1 M H_2_O_2_ were successively added to 80 μL of the released supernatant. After reacting for 15 min, color change was monitored by the naked eye and the UV–vis absorption spectrum was determined using the spectrophotometer.

## 3. Results and Discussion

### 3.1. Principle of Triple-Mode Immunoassays

**Scheme 1:**
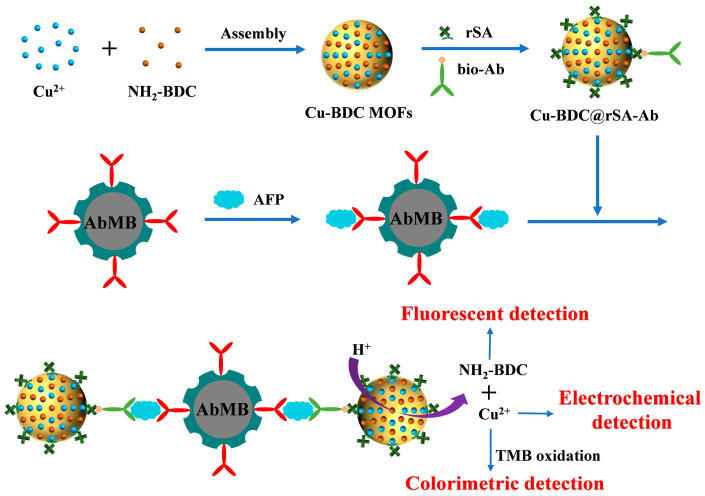
A schematic representation of the fluorescent–electrochemical–colorimetric triple-mode immunoassays.

In this work, the recognized cancer biomarker AFP was determined as the model [29,30]. Sandwich-like immunoreactions were performed on capture antibody-covered magnetic beads (AbMB) to achieve signal-on triple-mode immunoassays of AFP with low background signals. The detection principle is depicted in Scheme 1. Cu-BDC MOFs prepared by the assembly of Cu^2+^ and NH_2_-BDC were used as multifunctional sensing materials, which have the following advantages: (1) the simple preparation and modification processes of MOFs, (2) the strong fluorescence signal of the NH_2_-BDC ligand, and (3) well-defined electrochemical signal and high catalytic activity of Cu^2+^. Site-specific and oriented immobilization of recognition elements on the solid surface can facilitate the intermolecular interactions. Peptides or proteins with multiple imidazole groups in the histidine amino acid residues can be anchored on the metal surface through the coordination interactions of unsaturated metal sites. For this consideration, many commercial proteins are prepared by recombinant techniques to endow them with hexahistidine (His_6_) tags for convenience of separation and purification. The non-covalent and strong interactions between the unsaturated metal sites and the His_6_ tags have been used for the site-specific immobilization or labeling of proteins, thereby promoting the development of various biosensors [31,32]. It has been suggested that the His_6_-tagged proteins can be immobilized on the surface of MOFs by metal coordination interactions [28]. Such interactions have facilitated the modification of MOFs with biorecognition elements for the design of various biosensors. Therefore, recombinant protein streptavidin (rSA) was modified on the surface of MOFs through the metal coordination interactions, which can attach biotinylated antibody (bio-Ab) and eliminate non-specific adsorption (Scheme 1). In the presence of AFP, Cu-BDC@rSA-Ab conjugates were captured by AbMB through the antigen–antibody interactions and then decomposed by an acidic solution to release NH_2_-BDC and free Cu^2+^. Thus, the target was readily determined with triple-mode signals based on the fluorescent titration of NH_2_-BDC, the electrochemical reduction in Cu^2+^, and the Cu^2+^-catalytic oxidation of TMB. The proposed fluorescent–electrochemical–colorimetric triple-mode biosensing platform shows great potential for different applications, since it can be easily extended to other bioassays by changing the targets and receptors.

### 3.2. Characterization of Cu-BDC MOFs

**Figure 1:**
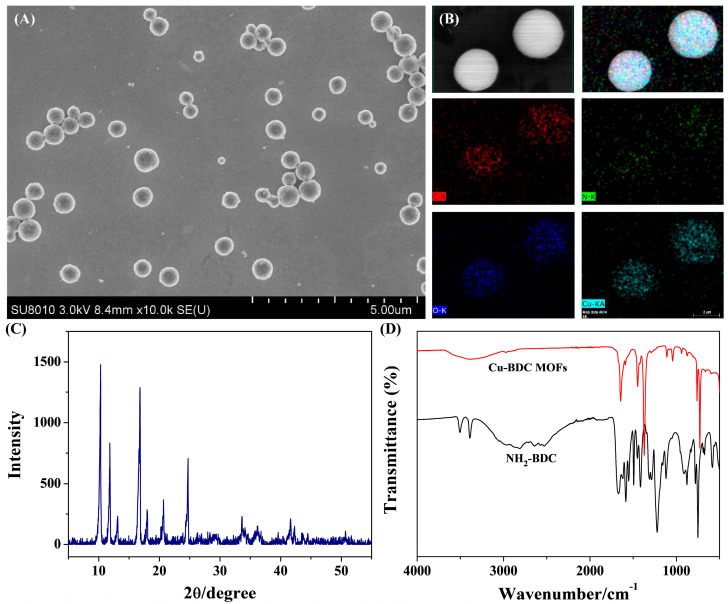
SEM image (**A**), EDS elemental mappings (**B**), XRD pattern (**C**), and FT-IR spectrum (**D**) of Cu-BDC MOFs.

**Figure 2:**
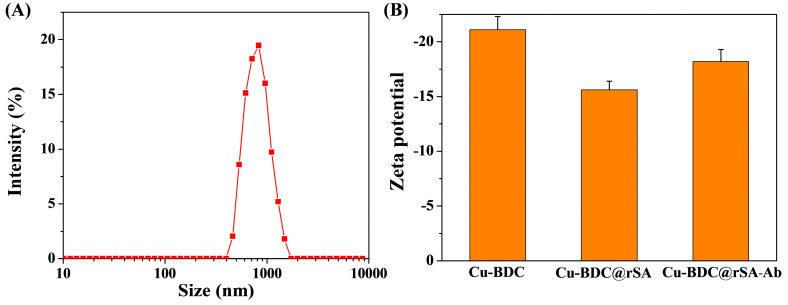
DLS (**A**) and ζ-potential (**B**) of Cu-BDC MOFs.

As presented in Figure 1A, the prepared Cu-BDC showed a sphere-shaped morphology. Their elemental composition was analyzed by EDS. As shown in Figure 1B, the elemental mappings of Cu-BDC displayed the distribution of C, N, O, and Cu elements, demonstrating that the Cu-BDC MOFs were successfully prepared. The structure of Cu-BDC MOFs was characterized by XRD. As shown in Figure 1C, the diffraction peaks were in good agreement with those reported in the literature [33], indicating that the prepared Cu-BDC MOFs exhibit a high degree of crystallinity. In addition, the FT-IR spectra of Cu-BDC and pure NH_2_-BDC samples were collected. As shown in Figure 1D, the stretching vibration peaks of NH_2_-BDC from the O–H and H bonding in the band of 2500~3000 cm^−1^ disappeared after the coordination with Cu^2+^, indicating Cu^2+^ was coordinated by the carboxyl groups in NH_2_-BDC. In this work, the Cu-BDC@rSA hybrids were used as the signal labels of immunoassays. To prove that rSA was modified on the MOF surface, the size and ζ-potential changes in Cu-BDC were monitored. It was noticed that the size of Cu-BDC MOFs measured by DLS (Figure 2A) was larger than that observed by SEM. This is understandable, since the DLS size is derived from the hydration radius but not the real diameter of particles. It was found that the average size of Cu-BDC increased from 842 to 863 nm after the modification of rSA, which was accompanied by the change in ζ-potential from −21.1 to −15.6 mV (Figure 2B). The results confirmed that rSA was successfully modified on the surface of Cu-BDC MOFs. In this work, the detection antibody of bio-Ab was conjugated onto the MOF surface through the classical avidin–biotin interaction. We found that the modification of bio-Ab did not induce a significant change in the size of Cu-BDC@rSA, but the ζ-potential changed from −15.6 into −18.2 mV.

### 3.3. Feasibility of Triple-Mode Immunoassays

**Figure 3:**
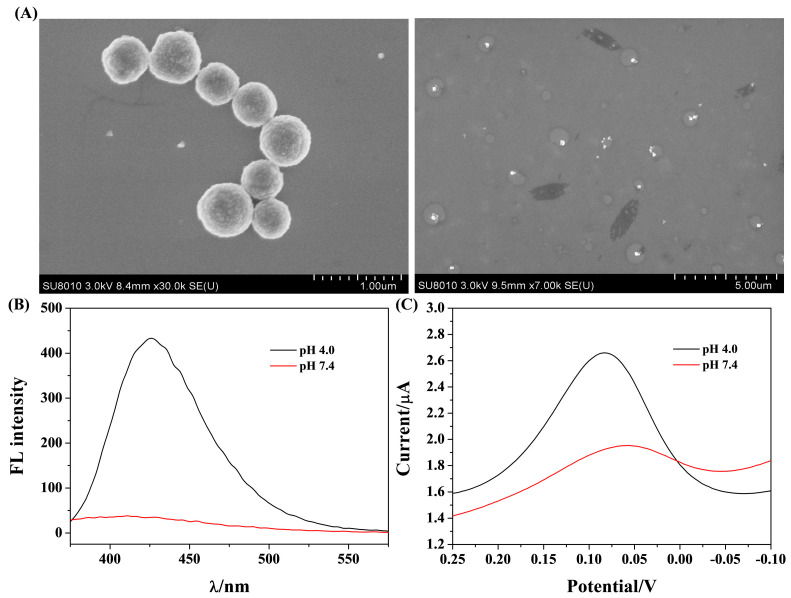
(**A**) SEM images of Cu-BDC MOFs before (left) and after (right) decomposition by 10 mM HCl. Fluorescence spectra (**B**) and DPV curves (**C**) of HCl-treated Cu-BDC in pH 4.0 acetic acid and pH 7.4 phosphate buffer, respectively.

To investigate the feasible applicability of the multifunctional MOFs in fluorescent–electrochemical–colorimetric triple-mode biosensing platforms, three key issues should be elucidated: (1) whether the Cu-BDC MOFs can be decomposed under an acidic condition to release Cu^2+^ and NH_2_-BDC, (2) whether the released Cu^2+^ and NH_2_-BDC can be determined by electrochemical, colorimetric, and fluorescent assays, and (3) whether Cu^2+^ and NH_2_-BDC interfere with each other in their respective assays. As shown in Figure 3A, the morphology of Cu-BDC MOFs did not alter after the modification of rSA. However, no sphere-shaped MOFs were found when they were incubated with 10 mM HCl, suggesting that the Cu-BDC MOFs could be decomposed under an acidic condition. The HCl-treated Cu-BDC showed a fluorescence signal at 428 nm and a reduction peak at 0.08 V (black curve in Figure 3B,C), which was ascribed to NH_2_-BDC and free Cu^2+^, respectively. In addition, we found that the fluorescence intensity and peak current of the released NH_2_-BDC and Cu^2+^ in an acidic acetic acid buffer solution were higher than the respective values in a neutral phosphate-buffer solution (red curve). This result is understandable, since the fluorescence of NH_2_-BDC could be quenched by Cu^2+^ at the neutral pH due to the ligand–metal charge transfer (LMCT) and the electrochemical signal of Cu^2+^ can be reduced due to the Cu^2+^-phosphate coordination interaction. It is noted that NH_2_-BDC and Cu^2+^ did not interfere with each other in their respective fluorescent and electrochemical signals, and the TMB-based colorimetric assays are usually conducted under acidic conditions. The released NH_2_-BDC and Cu^2+^ from the decomposition of MOFs in the following triple-mode assays were determined in an acetic buffer. The well-defined reduction peak of Cu^2+^ and the excellent fluorescent signal of NH_2_-BDC implied that the Cu-BDC MOFs can be used as the signal labels of multimode immunoassays. In addition, we found that the HCl-treated Cu-BDC could catalyze the oxidation of TMB by H_2_O_2_, which can endow the triple-mode assays with more information and visual self-validation.

### 3.4. Sensitivity of Triple-Mode Immunoassays

**Figure 4:**
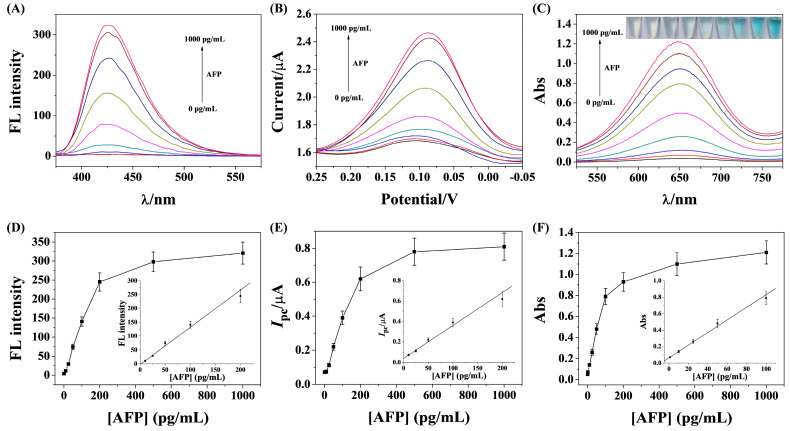
Fluorescence spectra (**A**), DPV curves (**B**), and UV-vis spectra (**C**) for the detection of different concentrations of AFP. The inset in (**C**) depicts the solution color change in the colorimetric mode. The calibration plots of DPV current (**D**), fluorescence intensity (**E**), and absorbance intensity (**F**) versus AFP concentration. The inserts show the linear portions of the calibration curves.

To evaluate the sensitivity of this strategy, AFP was determined based on the proposed fluorescent–electrochemical–colorimetric triple-mode immunoassays. As shown in Figure 4A–C, the triple signals are proportional to AFP concentrations, indicating more AFP can facilitate the capture of more Cu-BDC MOFs and lead to the release of more NH_2_-BDC and Cu^2+^. For the fluorescent immunoassay, the linear equation between the fluorescence signal of NH_2_-BDC and AFP concentration within the range of 10–200 pg/mL is FL = 1.3[AFP] (pg/mL) − 2.3 (R^2^ = 0.988) (Figure 4D). For the electrochemical immunoassay, the peak current was linearly related to AFP concentration, ranging from 10 to 200 pg/mL with a linear equation of *I*_pc_ = 0.003[AFP] (pg/mL) + 0.04 (R^2^ = 0.979) (Figure 4E). For the colorimetric immunoassay, the linear relationship between the absorbance intensity and AFP concentration ranging from 1 to 100 pg/mL is Abs = 0.008[AFP] (pg/mL) + 0.065 (R^2^ = 0.997) (Figure 4F). The detection limits of the three methods were estimated to be 10, 10, and 1 pg/mL by determining the minimum target concentration that can be apparently distinguished with that of the background control. Compared with the fluorescence assay, the blank control for the electrochemical and colorimetric mode showed a significant background signal, which should be attributed to the free Cu^2+^ resulting from the buffer or atmospheric dust, since the DPV and TMB-based methods are highly sensitive for the detection of Cu^2+^. The fluorescent–electrochemical–colorimetric triple-mode immunoassays exhibited lower detection limits and wider linear ranges through the release-based signal amplification strategy. The sensitivity is comparable to other immunoassay platforms based on the stimuli-responsive release of signaling molecules or ions, including coumarin 153-loaded polystyrene particles for C-reactive protein detection (4.9 ng/mL) [34], thymolphthalein-loaded mesoporous silica nanoparticles or metal-polydopamine frameworks for colorimetric immunoassays of PSA (0.36 pg/mL) or AFP (2.3 pg/mL) [35,36], self-assembled TMB nanoparticles for colorimetric immunoassays of interleukin-6 (1.92 pg/mL) [37], self-assembled tetra(4-carboxyphenyl)porphyrin (TCPP) nanoparticles for fluorescent immunoassays of IgG (2.05 pM) [38], and fluorescein-loaded gold nanoflower for fluorescent–colorimetric dual-mode immunoassays of AFP (29 fg/mL) [39]. In particular, the colorimetric assay exhibited high sensitivity and simplicity, and the detection results of the three methods could be self-validated with each other. The analytical performances for AFP detection were comparable to or even better than others achieved by a single-mode immunosensing platform (Table 1). In addition, the methods are sensitive, in contrast to the previously reported dual-mode immunoassays of protein biomarkers (Table 2). The proposed triple-mode sensing platform shows great potential for different applications, since it can be easily extended to other bioassays by changing the targets and receptors. Although the proposed triple-mode biosensors show high throughput, provide multilevel detection data and reliable results, and obtain a greater amount of available information, they require three different devices to collect the detection signals. This will increase the difficulty in clinical trials when taking into account assay time, detection cost, and operation steps. Thus, portable devices with on-site detection are required to meet point of-care test detection by directly converting the target concentrations into readable multiple signals within the same reaction system. In addition, although MOFs are believed to be the next-generation sensing materials, the large-scale environment-friendly production of MOFs with multi-functionality and high stability in an aqueous solution should be considered for biosensing applications [40,41].

### 3.5. Selectivity and Recovery

**Figure 5:**
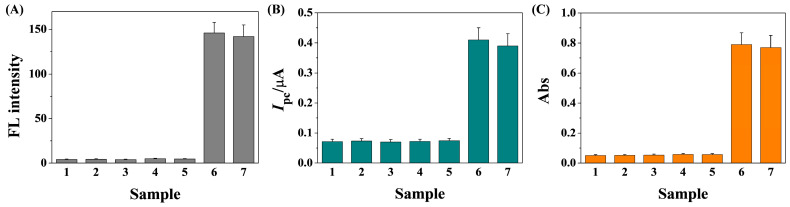
Selectivity of the fluorescent–electrochemical–colorimetric triple-mode immunoassays, shown in Panel (**A**–**C**), respectively. Samples 1~7: buffer blank, BSA, thrombin, CEA, PSA, AFP, and the mixture of 1~6. The concentration of AFP was 100 pg/mL and that of other proteins was 1 ng/mL.

Selectivity is a key factor in evaluating the application of biosensors in complex biological matrixes. As shown in Figure 5, serum protein BSA, protease thrombin, and different protein biomarkers (CEA, PSA, and AFP) were selected as the possible interferents to evaluate the selectivity of the proposed triple-mode immunoassays. It was found that only AFP caused large fluorescent, electrochemical, and colorimetric signals, although the interferents were tested at the concentration of 10-fold higher than that of the target. In addition, the presence of the interferences did not affect the detection of AFP. The results suggest that the proposed triple-mode immunoassays possess satisfactory selectivity and good anti-interference ability.

To further investigate the feasibility of the triple-mode sensing platforms for target detection in complex biological samples, a commercial bovine serum was analyzed with negligible effect of original AFP. Three concentrations of AFP standard samples were spiked in the serum, and then the fluorescent, electrochemical, and colorimetric signals of the triple-mode immunoassays were recorded. The final concentrations were calculated based on the corresponding linear equations. As shown in Table 3, the contents found were close to the added values, and the recoveries varied from 94.4% to 107.2% with relative standard deviations (RSDs) lower than 11%. This indicated that the recoveries obtained from the triple-mode immunoassays were acceptable. In addition, the quantification results were convincing, since the multimode detection methods can be mutually validated.

## 4. Conclusions

In summary, a triple-mode immunoassay platform was developed by using multifunctional Cu-BDC MOFs as the signal labels. Based on the strong fluorescent signal of NH_2_-BDC, the well-defined electrochemical signal of Cu^2+^, and the high catalytic activity of Cu^2+^ toward TMB oxidation, the developed fluorescent–electrochemical–colorimetric triple-mode biosensing platform exhibited excellent performances for the detection of AFP with wide linear ranges and low detection limits. In addition, the proposed triple-mode immunoassays possess improved detection accuracy and reliability through mutual validation in three detection modes, showing great potential in disease diagnosis and bioanalysis. Different detection modes can compensate for each other’s limitations. However, there still remains room for improvement in the development of triple-mode immunoassays to meet the demand of practical sensing applications. For example, multimode portable devices should be developed to enable efficient multiple-signal detection for practical applications, and advanced nanomaterials with multi-functionality, uniform morphology, and low toxicity should be explored to increase the detection accuracy and environmental friendliness of biosensors. Efforts are being made in our group to explore multiple-mode signal labels with high stability, low production cost, and excellent environmental friendliness.

## Figures and Tables

**Table 1:** Analytical performances of different sandwich-like immunoassay methods for the detection of AFP.

Method	Signal Label	Signal Output	Linear Range (ng/mL)	Detection Limit (pg/mL)	Ref.
FL	ALP/Si NPs	Enzyme catalysis	1~60	1000	[42]
FL	AOX–AuNPs/Mn^2+^	Enzyme catalysis	0.05~20	17.3	[43]
FL	ALP/calcein-Ce^3+^	Enzyme catalysis	1~40	41	[44]
FL	AgNPs/TPE-4TA	Signal probe	0.01~5000	42	[45]
FL	CuO NPs/CdS QDs	Signal probe	1~80	450	[46]
FL	CuO NPs/CdS QDs/ENFFs	Signal probe	0.01~200	8.3	[47]
FL	bio-PyFNPs	Signal probe	0.001~2.5	0.5	[48]
EC	CdS QDs	Electrooxidation	0.001~1	0.11	[49]
EC	ADM@AuNPs@SiO_2_	Electrooxidation	0.0005~75	0.17	[50]
EC	Thi-Au-Fe_3_O_4_@ZIF-8	Electroreduction	Not reported	0.003	[51]
EC	MnO_2_ nanosheets	Electrocatalysis	0.005~100	1	[52]
EC	CNSs@AuNPs@Zn^2+^	Electrocatalysis	0.010~80	2.6	[53]
EC	DPNs	Electrocatalysis	0.1~50	40	[54]
Color	MnO_2_ NPs	Nanocatalysis	6.25~400	22	[55]
Color	FePor-TFPA-COP	Nanocatalysis	0.005~100	1	[56]
Color	ADH	Enzyme catalysis	Not reported	1	[57]
Color	ALP	Enzyme catalysis	1250~10,000	148	[58]
Color	β-gal	Enzyme catalysis	0.1~100	80	[59]
Color	MPDA@TP	Color indicator	0.01~1	2.3	[36]

Abbreviations: FL, fluorescence. EC, electrochemical. ALP, alkaline phosphatase. AOX, ascorbate oxidase. TPE-4TA, tetrazole-tagged luminogen. ENFFs, electrospun nanofibrous films. bio-PyFNPs, biotinylated pyrene–phenylalanine nanoparticles. QDs, quantum dots. CNSs, carbon nanospheres. AuNPs, gold nanoparticles. DPNs, dendritic Pt nanoparticles. ADM, adriamycin. Thi, thionine. ZIF-8, zeolitic imidazolate framework-8. MnO_2_ NPs, manganese dioxide nanoparticles. FePor-TFPA-COP, iron porphyrin-based covalent organic polymer. ADH, halcoholde hydrogenase. MPDA@TP, thymolphthalein-modified metal–polydopamine framework. β-gal, β-galactosidase.

**Table 2:** The analytical performances of colorimetric–electrochemical and colorimetric–fluorescent dual-mode immunoassays for the detection of protein biomarkers.

Method	Signal Label	Target	Linear Range	Detection Limit	Ref.
Colorimetric–Electrochemical	PBNPs	TRX1	10~50 ng/mL	9 and 6.5 ng/mL	[60]
	PBNPs	IgG	0.5~10 µg/mL	34 ng/mL	[61]
	ALP	CA125	5~1000 and 50~1000 U/mL	1.3 and 40 U/mL	[62]
	HRP-Ppy NPs	PSA	0.001~40 ng/mL	0.8 and 0.7 pg/mL	[63]
	uPtNZs	GA	0.01~5 and 0.005~10 mg/mL	9.2 and 3.8 µg/mL	[64]
	H-AuNPs	BNP	0.001~0.2 and 5~25 ng/mL	0.03 and 80.3 pg/mL	[65]
Colorimetric–Fluorescent	Fe-MOFs	PSA	1~20 and 0~15 ng/mL	180 pg/mL	[66]
	CsPbBr3 NCs	PSA	0.1~15 and 0.01~80 ng/mL	290 and 81 pg/mL	[67]
	porous Pd NPs	NMP22	0.001~0.5 ng/mL	0.35 and 0.31 pg/mL	[68]
	nanoceria	cTnI	0.001~10 ng/mL	227 and 413 fg/mL	[69]
	ALP	cTnI	0.2~80 and 0.05~4 ng/mL	60 and 15 ng/mL	[70]
	ALP	PSA	0.02~28 and 0.02~20 ng/mL	4.1 and 9.6 pg/mL	[71]
	Au@CD	ferritin	1~160 and 1~120 ng/mL	20 and 64 ng/mL	[72]
	GOx	PSA	0.005~20 ng/mL	2.3 and 0.84 ng/mL	[73]
	QDs/ZnS NSs	AFP	0.05~12 ng/mL	7 and 10 pg/mL	[74]
	AuNF@Fluorescein	AFP	5~5000 and 0.01~10 pg/mL	17.7 and 0.029 fg/mL	[39]

Abbreviations: PBNPs, Prussian blue nanoparticles. TRX1, thioredoxin 1. IgG, immunoglobulin G. ALP, alkaline phosphatase. CA125, carbohydrate antigen 125. HRP, horseradish peroxidase. Ppy NPs, polypyrrole nanoparticles. PSA, prostate-specific antigen. uPtNZs, urchin-like Pt nanozymes; GA, glycated albumin. H-AuNPs, hemin-modified gold nanoparticles; BNP, brain natriuretic peptide. Fe-MOFs, Fe(III)-containing metal–organic frameworks. CsPbBr3 NCs, CsPbBr3 perovskite nanocrystals. NMP22, nuclear matrix protein 22. cTnI, cardiac troponin I. Au@CD, gold@carbon dot. GOx, glucose oxidase. QDs/ZnS NSs, ZnS nanospheres modified with CdTe quantum dots; AFP, alpha-fetoprotein. AuNF, gold nanoflower.

**Table 3:** The results for the assays of AFP in serum samples.

Added (pg/mL)	Found by Fluorescence (pg/mL)	Found by DPV (pg/mL)	Found by UV-vis (pg/mL)
25.0	25.6 ± 2.5	24.5 ± 2.6	26.8 ± 2.8
50.0	48.8 ± 4.6	47.2 ± 4.8	50.2 ± 5.2
100.0	103.5 ± 9.8	101.5 ± 10.2	104.5 ± 10.8

## Data Availability

Data are contained within the article.
